# The NKG2D/NKG2DL Axis in the Crosstalk Between Lymphoid and Myeloid Cells in Health and Disease

**DOI:** 10.3389/fimmu.2018.00827

**Published:** 2018-04-23

**Authors:** Ana Stojanovic, Margareta P. Correia, Adelheid Cerwenka

**Affiliations:** ^1^Innate Immunity (D080), German Cancer Research Center (DKFZ), Heidelberg, Germany; ^2^Department of Immunobiochemistry, Medical Faculty Mannheim, University of Heidelberg, Mannheim, Germany

**Keywords:** NKG2D, NKG2D ligands, myeloid cells, natural killer cells, NKG2D+ T cells

## Abstract

Natural killer group 2, member D (NKG2D) receptor is a type II transmembrane protein expressed by both innate and adaptive immune cells, including natural killer (NK) cells, CD8+ T cells, invariant NKT cells, γδ T cells, and some CD4+ T cells under certain pathological conditions. NKG2D is an activating NK receptor that induces cytotoxicity and production of cytokines by effector cells and supports their proliferation and survival upon engagement with its ligands. In both innate and T cell populations, NKG2D can costimulate responses induced by other receptors, such as TCR in T cells or NKp46 in NK cells. NKG2D ligands (NKG2DLs) are remarkably diverse. Initially, NKG2DL expression was typically attributed to stressed, infected, or transformed cells, thus signaling “dysregulated-self.” However, many reports indicated their expression under homeostatic conditions, usually in the context of cell activation and/or proliferation. Myeloid cells, including macrophages and dendritic cells (DCs), are among the first cells sensing and responding to pathogens and tissue damage. By secreting a plethora of soluble mediators, by presenting antigens to T cells and by expressing costimulatory molecules, myeloid cells play vital roles in inducing and supporting responses of other immune cells in lymphoid organs and tissues. When activated, both macrophages and DCs upregulate NKG2DLs, thereby enabling them with additional mechanisms for regulating lymphocyte responses. In this review, we will focus on the expression of NKG2D by innate and adaptive lymphocytes, the regulation of NKG2DL expression on myeloid cells, and the contribution of the NKG2D/NKG2DL axis to the crosstalk of myeloid cells with NKG2D-expressing lymphocytes. In addition, we will highlight pathophysiological conditions associated with NKG2D/NKG2DL dysregulation and discuss the putative involvement of the NKG2D/NKG2DL axis in the lymphocyte/myeloid cell crosstalk in these diseases.

## The Natural Killer Group 2, Member D (NKG2D) Receptor

Natural killer group 2, member D is a type II transmembrane protein with a C-type lectin-like extracellular domain, expressed on the cell surface as a disulfide-linked homodimer. The receptor possesses a short intracellular tail with charged amino acid residues that enable its association with the adaptor molecules DAP10 and/or DAP12, which is essential for NKG2D surface expression (1–3). In mice, alternative splicing of NKG2D results in two distinct isoforms: NKG2D-short and NKG2D-long. The NKG2D-short isoform can associate with both DAP10 and DAP12, whereas the NKG2D-long isoform associates only with DAP10 (2). Freshly isolated naïve natural killer (NK) cells express only the NKG2D-long isoform that forms complexes with DAP10. Activated NK cells express both NKG2D isoforms and can therefore pair with both adapters (2). However, human NK cells only express the NKG2D-long isoform, and consequently, NKG2D appears to only use DAP10 for signal transduction. NKG2D triggering induces cytotoxic responses, secretion of cytokines and chemokines, and supports proliferation and survival of responding effector cells (4).

The NKG2D receptor gene (*KLRK1*, killer cell lectin-like receptor K1) was first described in 1991 in human NK cells (5). Since then, several other lymphocytes have been shown to express NKG2D, namely, all αβCD8+ T cells in human, activated, but not naïve, αβCD8+ T cells in mice, γδ T cells, invariant NKT (*i*NKT) cells, and some CD4+ T cells under certain pathological conditions. More recently, NKG2D was detected on the surface of innate lymphoid cells (ILCs), including helper ILC1 and ILC3 (6–8). Importantly, those different NKG2D-expressing immune cell types reside in distinct morphological sites, such as lymphoid organs, skin, and epithelial and sub-epithelial tissues. Indeed, NKG2D-expressing cells and upregulation of NKG2D ligands (NKG2DLs) were shown to play important roles in inflammation, anti-tumor, and anti-viral responses in different organs (9, 10). In addition, the pathophysiology of several autoimmune conditions, as well as acute and chronic allograft rejection, involved dysregulated expression or/and activation of NKG2D and its ligands (11–17).

Expression of NKG2D on immune effector cells is regulated by cell activation and microenvironmental factors. In NK cells, which constitutively express NKG2D, the cytokines IL-2, IL-12, and IL-15 were shown to upregulate, while IL-21, IFN-γ, and TGF-β negatively regulated NKG2D surface expression (18). The triggering of the NKG2D receptor in mouse and human NK cells can induce effector functions, such as cytotoxicity and cytokine production. However, several studies revealed that pre-activation with cytokines, such as IL-2 or IL-15, was required to trigger NK cell responses upon NKG2D engagement (19, 20). This feature might have evolved to assure NKG2D activation only in cases when potential “danger signals” are present in the context of inflammation, characterized by the production of inflammatory cytokines. In addition, NKG2D can act as a costimulatory molecule, able to induce cytolytic activity in resting NK cells, when cotriggered with other activating receptors, such as NKp46 or 2B4 (9). Several mechanisms account for cytokine-mediated priming of NKG2D responsiveness, such as IL-2-mediated activation of mTORC1 and upregulation of amino acid transporters (20), or IL-15-induced phosphorylation of the adaptor molecule DAP10 (19) and activation of cytosolic phospholipase A_2_ accompanied by production of arachidonic acid (21). In human NK cells and resting mouse NK cells, which express the NKG2D-long isoform, the signal is transduced *via* DAP10 and propagates through Grb2/Vav and the PI3K pathway, similar to the costimulatory molecule CD28 (22), which might explain the need for additional signals for cell activation. In mouse, activation induces changes in *Nkg2d* mRNA alternative splicing, leading to the expression of the NKG2D-short isoform. Its coupling to the ITAM-bearing adaptor DAP12, which recruits and activates signaling *via* Syk and ZAP70 protein kinases, has been implicated in the triggering of the NKG2D-short isoform without the need of cytokine “priming” or coreceptor activation (22). These results signify that NKG2D function on NK cells depends on the NK cell activation status and tightly correlates with the presence of additional microenvironmental signals, such as cytokines or ligands of other receptors expressed on target or neighboring cells. Therefore, it is not surprising that NKG2D-deficient mice do not show a major phenotype until crossed to TRAMP or Eμ-Myc mice, which spontaneously develop prostate cancer and lymphoma, respectively (23).

## NKG2DLs: Expression and Regulation

Although NKG2D is largely not polymorphic (only two alleles with a single aa difference exist in human) and shows strong evolutionary conservation with ~70% sequence identity between mouse and human, this receptor is able to bind a broad range of stress-induced ligands that, in contrast, show a high degree of polymorphism (24, 25). In the context of transplantation, polymorphic NKG2DLs can cause donor–recipient incompatibility and lead to allograft rejection by inducing the formation of antibodies directed against NKG2DL epitopes and complement-dependent cytotoxicity (26–29).

NKG2D ligands comprise several MHC class I-like molecules, which include murine UL16-binding protein-like transcript 1 (MULT1), retinoic acid early transcripts α-ε (RAE-1 α-ε) and H60 a-c in mice, and MHC class I-related genes A and B (MICA and MICB) and UL16-binding protein (ULBP) family in human. All NKG2DLs have α1α2 domains responsible for binding to the NKG2D receptor, however, only low sequence similarity can be observed between various ligands, indicating a significant level of variability. It was proposed that the variability of these ligands increased with coevolution with pathogens, allowing their differential expression patterns among cells and tissues, distinct intracellular trafficking, and differential affinity for the NKG2D receptor, which might influence the strength of the delivered signal (24).

NKG2D ligand expression is most frequently associated with infection, cell stress, and transformation, thus alerting for “stressed- and damaged-self.” Distinct forms of cell stress can induce cell surface expression of NKG2DLs, including DNA damage, oxidative stress, heat-shock, or the ER stress response (30–35). For example, the p53 pathway, involved in the DNA damage response, was shown to strongly upregulate ULBP1 and ULBP2 at both mRNA and protein level. Similarly, heat-shock-induced transcription factor HSF1 can drive MICA promoter activation (36). Other transcription factors, including E2F, NF-kB, ATF4, the Sp-family, and AP-1, were also shown to be involved in NKG2DL mRNA transcription (36–38). Sauer et al. showed that histone acetylation and binding of acetyltransferases CBP and p300 to NKG2DL promoter regions increased NKG2DL expression by tumor cells (39), suggesting the importance of an open chromatin state in the regulation of NKG2DL expression. In addition, NKG2DL expression has been associated with viral infections, including CMV, influenza, hepatitis B, Epstein–Barr, and adenovirus (40), as well with as some bacterial infections (e.g., *E. coli, M. tuberculosis*) (24). Accordingly, triggering of toll-like receptors (TLRs), that sense microbial products, also induced NKG2DL expression in macrophages and dendritic cells (DCs) (41, 42). Certain viruses, such as HIV, engage the DNA damage pathway, while other viruses, such as MCMV, induce NKG2DL expression *via* PI3K-mediated activation (35).

NKG2D ligand expression is regulated at several levels (transcriptional, post-transcriptional, and post-translational) and depends on the cell type, its activation and metabolic state, and the microenvironmental context (35, 36). Cytokines, such as IFN-γ, IFN-α, and TGF-β, were reported to regulate NKG2DL expression on mouse and human cell lines (10). NKG2DL surface expression is further controlled by miRNAs and mRNA-binding proteins that target NKG2DLs at the transcript level (43–45), by alternative splicing (37), and by Ub-mediated degradation of NKG2DL protein(s) (46, 47). In various cell types and tissues, NKG2DL RNA transcripts were detected in the absence of protein (40). In gut and bronchial epithelia, ligands can be stored intracellularly and transferred to the cell surface upon stimulation (40). In addition, NKG2DLs can be delivered to effector cells *via* exosome secretion, which represents a potent mechanism exploited by cancer cells to downregulate NKG2D expression on effector cells (48). NKG2DL surface expression can be downregulated by proteolytic shedding at the plasma membrane; however, this process depends on the type of ligand, and it seems to be regulated by the microenvironment (49). For example, elevated serum levels of soluble MIC proteins were detected in patients with different types of cancer and correlated with unfavorable prognosis. Soluble MIC was shown to induce NK and CD8+ T cell dysfunction by inducing loss of the CD3ζ signaling molecule, or by decreasing NKG2D surface expression through endocytosis and degradation upon its engagement (50). By contrast, soluble MULT1, a high affinity mouse NKG2DL, was shown to counteract NK cell desensitization and to cause NK cell activation and tumor rejection *in vivo* (51). Opposite to cancer, in autoimmune disease, although increased levels of MIC proteins in serum have been detected, NKG2D expression was not affected in the analyzed patients (50).

Although most frequently associated with infection, cellular stress, and transformation, NKG2DLs are also detected on certain cell types in the absence of pathophysiological conditions [reviewed in Ref. (40)]. These include subsets of uncommitted thymocytes, activated T and myeloid cells, class-switching B cells, regulatory T cells, myeloid-derived suppressor cells (MDSCs), bone marrow stromal cells, committed myelomonocytic progenitors, pluripotent mesenchymal cells, and epithelial cells of the respiratory and gut mucosa. Other cells, such as myoblasts, hepatocytes, neurons, and mouse embryonic cells, were also reported to express NKG2DLs, but their regulation and function on these cells is less clear. In the case of hematopoietic cells, it has been reported that NK cells can eliminate activated immune cells in an NKG2D-dependent manner, thereby restraining T cell responses or excessive inflammation mediated by myeloid cell activation. In the thymus, NKG2D might be involved in the regulation of the T cell repertoire (40), while at the epithelial barriers, NKG2D/NKG2DL expression can be linked to continuous presence of commensal microbiota and constitutive state of low-grade, the so-called physiological inflammation (52). Recently, Thompson and colleagues reported that endothelial cells in lymph nodes constitutively expressed Rae-1, whose engagement at steady state led to downmodulation of NKG2D expression and function in circulating NK cells (53). Human activated NK cells can themselves upregulate certain ULBP family members after culture with the cytokines IL-12/15/18 (54). In this case, intercellular NKG2D activation led to ADAM17-mediated release of TNF-α, thus promoting NK cell cytokine release. By contrast, NKG2DLs expressed on NK cells from diabetic NOD mice were postulated to negatively regulate expression of the NKG2D receptor (55). Moreover, NK cells could acquire NKG2DLs *via* trogocytosis upon interaction with target cells, which led to their fratricide, thereby resulting in downmodulation of the immune response (56).

## Expression of NKG2DLs by Myeloid Cells

**Figure 1:**
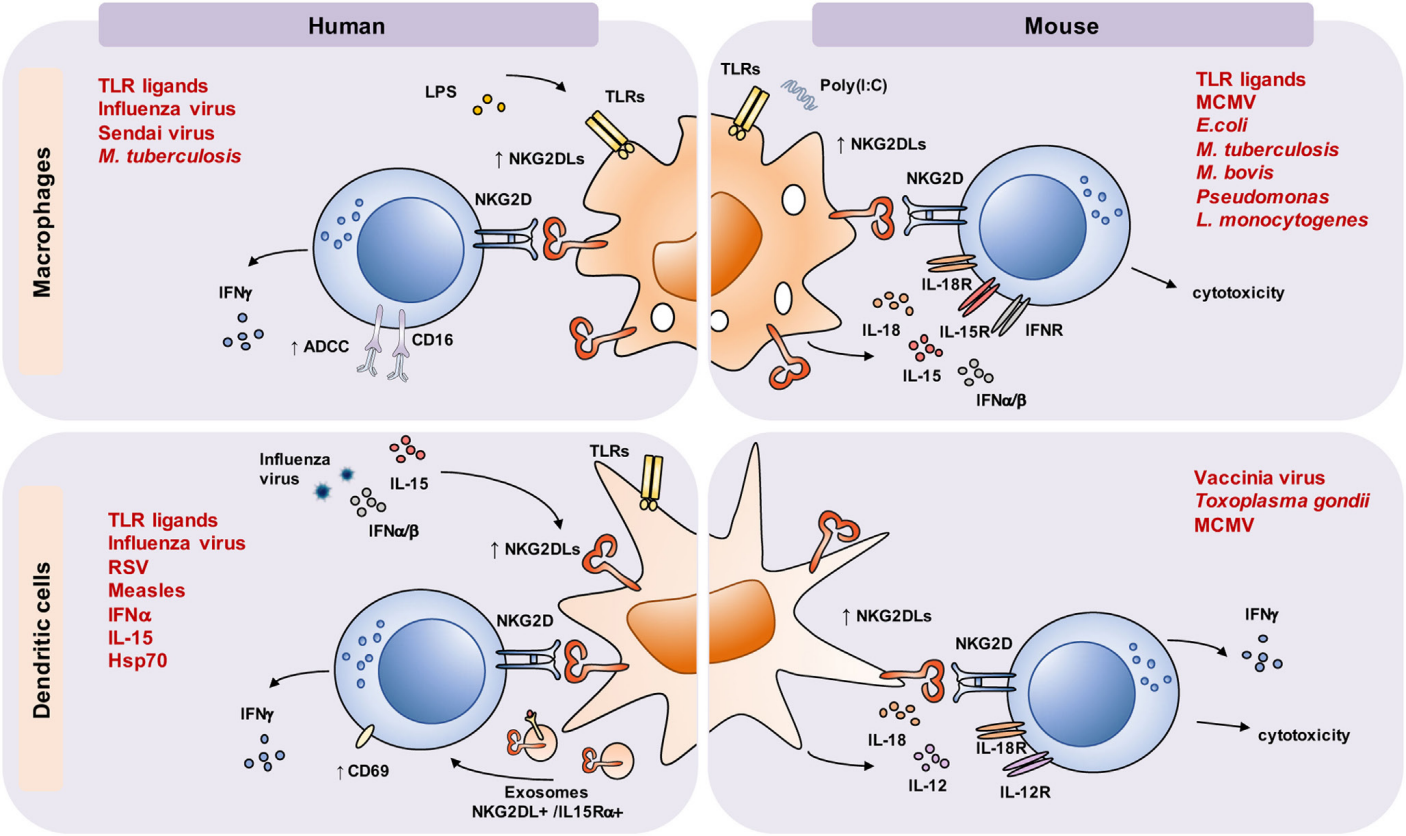
Macrophages and dendritic cells (DCs) activated by toll-like receptor (TLR) ligands, cytokines, viral or bacterial infection upregulate NKG2D ligands (NKG2DLs) and regulate natural killer (NK) cell effector responses. In both mouse and human, TLR activation and viral and bacterial infection were shown to upregulate NKG2DLs on macrophages and DCs. In addition, cytokines produced by myeloid cells upon infection, such as IL-15 or type I IFNs, can also induce NKG2DL expression on DCs and, importantly, increase natural killer group 2, member D (NKG2D) expression on interacting lymphocytes. Induced NKG2DLs interact with NKG2D expressed by NK cells and lead to their activation, resulting in secretion of IFN-γ, cytotoxicity, CD69 upregulation, and increased killing of antibody-coated cells by antibody-dependent cellular cytotoxicity (ADCC). NKG2DLs and IL-15Rα/IL-15 can also be delivered to NKG2D+ effector cells *via* exosomes. NK cell responses are further supported by soluble factors released by myeloid cells, including IL-12 and IL-18, which strongly synergize in IFN-γ induction. In turn, IFN-γ released by NK cells supports myeloid cell activation and release of soluble factors, creating a myeloid-lymphoid feedback activation loop. In some instances, activated NK cells can kill NKG2DL-expressing macrophages and DCs, thereby limiting their numbers, their responses, or improper stimulation. Abbreviations: RSV, respiratory syncytial virus; MCMV, mouse cytomegalovirus.

In both human and mice, various myeloid cell subsets were reported to express NKG2DLs (Figure 1). In many cases, NKG2DL induction on myeloid cells is a direct consequence of infection. For example, human macrophages upregulate NKG2DL expression upon influenza or Sendai virus infection (57). In infection settings, macrophages can directly sense pathogens using various innate immune receptors, some of which have been shown to directly regulate NKG2DL expression when triggered *in vitro*. Signaling *via* different TLRs can induce NKG2DLs in both monocytes and macrophages, but the nature of the induced ligand(s), the levels of their expression, and the magnitude of subsequent NKG2D-driven responses might vary. It is tempting to speculate that such differential responses were evolutionarily driven and tailored to fit the defense strategy against an invading pathogen. In primary human macrophages, TLR triggering induces MICA and MICB expression. Eissmann et al. showed that LPS not only induced MICA expression, but also decreased the levels of miRNAs involved in targeting MICA transcripts for degradation (58). In addition, while TLR4 signaling was required for *MICA* gene transcription, the ATM/ATR pathway, involved in the DNA damage response, controlled the expression of the MICA protein (58), highlighting the complexity of regulation of NKG2DL expression. The Davis lab also showed that TLR7/8, but not TLR3 ligation, induced both MICA and MICB, and that MICA expression correlated with macrophage activation, measured by the production of proinflammatory cytokines (58). Similar to macrophages, TLR-stimulated monocytes upregulated MICA, but not other NKG2DLs, along with CD80 and MHC class I and II (59). Besides MICA/B, monocytes and macrophages can express other NKG2DLs. TLR2-driven ULBP1 expression was reported on *M. tuberculosis* infected monocytes and alveolar macrophages, leading to their NKG2D-dependent lysis by NK cells (60). ULBP1 could also be induced on monocytes by growth factors, but not by the cytokines TNF-α, IL-1β, and IFN-α, or by the TLR4 ligand LPS (61).

Similar to human monocytes and macrophages, mouse macrophages were reported to express NKG2DLs in the context of infection, such as *M. tuberculosis* (62) or *P. aeruginosa* (63). Murine peritoneal macrophages expressed Rae-1, but not other NKG2DLs, upon stimulation *via* TLRs, both *in vitro* and *in vivo* (42). Exposure to G− (*E. coli*) and G+ (*S. aureus, L. monocytogenes*) bacteria, *Mycobacterium bovis* BCG or infection with CMV, all induced Rae-1 expression (42, 64), reinforcing that macrophage activation and pathogen recognition is linked to NKG2DL upregulation. Cytokines produced upon response to pathogens, such as TNF-α, type I IFNs, or IFN-γ, were not required for Rae-1 expression in these settings.

Besides infection, evidence exists of NKG2DL association with myeloid cell differentiation and acquisition of an activated effector phenotype. This is not surprising, as induction of NKG2DLs was correlated with cell proliferation (65) and DNA damage (30, 31), which might occur during effector responses in tissues. It was shown that human CD34+ hematopoietic stem cells (HSC) expressed low levels of NKG2DLs, which were increased upon commitment to the myeloid lineage (61, 66). In mice, HSC transplantation in irradiated animals gave rise to Gr1+ and CD11b+ cells expressing Rae-1 and H60 in the bone marrow (29). Similarly, myeloid cells with immunosuppressive function that accumulated in tumor-bearing mice were reported to express Rae-1 (51, 67). So far, the reason for NKG2DL expression by immature myeloid cells is unclear. In tumor settings, engagement of NKG2D on NK cells led to NK cell activation and cytolysis of Rae-1-expressing myeloid cells (67). Whether NK cells can control myeloid lineage differentiation *via* regulating the numbers of developing progenitors is, so far, elusive.

Of note, besides expressing NKG2DLs, there are some indications that activated macrophages could also upregulate the NKG2D receptor, although these observations remain controversial. Thioglycolate-induction *in vivo* or stimuli, such as LPS, type I IFNs, and IFN-γ, were shown to drive mRNA and protein NKG2D expression on peritoneal and bone marrow-derived macrophages, respectively. When stimulated with immobilized ligands or ligand-expressing cells, NKG2D+ macrophages produced nitric oxide and TNF-α (2, 68, 69), indicating that this system might enhance macrophage-mediated elimination of pathogens, pathogen-infected or tumor cells. Indeed, it was also suggested that crosstalk with certain NKG2DL+ tumor cells might contribute to macrophage activation and lower the threshold for their responses (70). However, this effect might also be exploited in tumor settings and facilitate cancer immune evasion. For example, Qian et al. detected NKG2D expression on Gr1+ CD11b+ myeloid cells accumulating in blood, spleen, and bone marrow of tumor-bearing mice. Blockade of NKG2D *in vitro* impaired IL-4 and IL-10 production by these cells, while *in vivo* neutralization reduced their accumulation in mice bearing Rae-1+ tumors (71).

In DCs, NKG2DL upregulation was also associated with activation caused by infection and/or cellular stress (Figure 1). In mouse, vaccinia virus infection induced Rae-1 (72), while pulse with *Toxoplasma gondii* lysates led to both Rae-1 and MULT1 expression on the DC surface (73). In infected human monocyte-derived DCs, influenza virus induced ULBP proteins (74). Other RNA viruses, such as respiratory syncytial virus, led to ULBP1 upregulation, while measles virus, as well as exposure to poly(I:C), upregulated ULBP2 (41). These data indicate that different mechanisms can be employed by DCs to induce expression of distinct NKG2DLs in response to various virus types. IFN-α, which is primarily produced during viral infections, was reported to induce MIC ligand expression on human DCs. A similar effect was attributed to IL-15, while stimulation with LPS, poly(I:C), CD40L, TNF-α, IL-12, and IL-18 did not affect NKG2DL expression (75, 76). MICA expression was also induced by Hsp70 (77), although bacterial products or other molecules might as well have contributed to this effect (78). TLR ligands, such as LPS and poly(I:C), were able to upregulate ULBPs on human monocyte-derived DCs (41). ULBP1 was also detected on mature DCs in T cell areas of lymph nodes *in situ*, in close proximity to NKG2D-expressing CD8+ T cells (79), indicating its possible involvement in T cell priming.

## The NKG2D/NKG2DL Axis in the Crosstalk of Myeloid Cells and NK Cells

Myeloid cells and innate lymphocytes form the first line of defense against invading pathogens. Their activation leads not only to the direct elimination of pathogens and their products, but also to the activation of proper adaptive immune responses. Moreover, it is becoming appreciated that these cells also respond to cues indicating tissue damage and, in addition to defense, orchestrate mechanisms of tissue repair (80). Since (i) both infection and damage were shown to upregulate NKG2DLs on myeloid cells, and (ii) factors produced in response to these events (by stroma, parenchymal, and immune cells) can induce or upregulate NKG2D on lymphocytes, an NKG2D-driven lymphocyte-myeloid cell crosstalk is expected to play an important role in these processes. Indeed, in many animal models, NKG2D genetic deletion or Ab-mediated blockade *in vivo* affected disease development and tissue repair, including tumor progression, autoimmunity, and wound healing (12, 13, 23, 65). However, the contribution of the individual cell populations to these observations remain not fully addressed. This particularly applies to cells expressing NKG2DLs, whose depletion or conditional targeting is experimentally challenging. In addition, the generation of NKG2DL-deficient mice is complicated by the existence of multiple ligands organized as gene families. However, recent success in creating Rae-1-deficient mice (51) opens the possibility to generate bone marrow chimeras and to address the contribution of Rae-1 expression, at least in the hematopoietic versus non-hematopoietic compartment, in disease settings *in vivo*.

In the case of myeloid cells, while their role in activating NKG2D-expressing cells *in vivo via* NKG2DLs remains largely unknown, *in vitro* data provide convincing evidence of an NK cell crosstalk with DCs and macrophages (Figure 1). For example, in the MCMV model, virus-infected mouse DCs play a crucial role in NK cell activation by both inducing IFN-γ release (through IL-12/18 production) and NK cell cytotoxic responses *via* IFNα and NKG2D engagement (81). In human *in vitro* system, it was shown that DCs infected with influenza virus supported CD69 upregulation and IFN-γ production by NK cells *via* NKG2D and NKp46 (74). In addition, it was shown that human DC-derived exosomes displayed ULBP1, together with IL-15Rα, on their surface and were able to promote NKG2D-dependent activation of NK cells (82). Also, MICA and MICB, induced on DCs upon IFN-α or IL-15 treatment, contributed to NK cell activation (75, 76). In these studies, the authors showed that type I IFNs and IL-15 induced MIC molecules on DCs, which was impaired in patients with chronic hepatitis C infection. Similarly, coculture of NK cells and DCs pulsed with *T. gondii* lysate increased DC IL-12 production and their ability to prime Ag-specific CD8+ T cell responses, which was impaired by NKG2D blockade (73). Besides mutual activation, the NK/DC crosstalk can also result in an NK cell cytolytic response, leading to DC elimination, which is considered essential in the regulation of the numbers and quality of the activated DCs and, consequently, the extent of the overall immune response. The outcome of the NK/DC crosstalk depends on the activation/maturation status of both interacting cell types and their relative abundance. Accordingly, human IL-2-activated NK cells increased mature DC responses, measured by the level of IL-12 release and ability to induce CD4+ T cell activation (83). In coculture with immature DCs (iDCs), activated NK cells were shown to support autologous DC maturation and activation at low NK/DC ratios, while increased numbers of NK cells resulted in iDC lysis (84). In these settings, cytotoxic NK cell responses were mainly mediated *via* the activating receptors NKp30 and DNAM-1 (85), although a partial contribution of NKG2D has also been observed (86). However, treatment of mature DCs with IL-10 induced their elimination by NK cells, which was mediated *via* NKG2D, while IL-10-treated iDCs resisted to NK-mediated cytotoxicity (87). As the authors of this study suggested, aberrant accumulation of iDCs in patients with chronic infections that are frequently associated with increased levels of IL-10 production, such as HIV, thus might be the consequence of IL-10-induced DC resistance to NK cell elimination (87). Remarkably, Schulz and colleagues showed that IL-10 also rendered autologous human macrophages susceptible to NK cell lysis, which involved NKG2D. In the presence of IL-10, NKG2D receptor expression on NK cells increased, while macrophages induced NKG2DL expression (88). Thus, IL-10 might exert its immunomodulatory pleotropic effect, not only *via* suppressing T cell responses, but also *via* inducing NK cell-mediated killing of activated myeloid cells, including antigen-presenting cells (APCs).

Similar to DCs, monocyte-derived macrophages were reported to express MICA and ULBP1-3 upon stimulation with LPS, rendering them susceptible to NK cell-mediated lysis (89). In alveolar macrophages, *M. tuberculosis* infection led to ULBP1 induction and their NKG2D-dependent lysis by NK cells (60). However, it was also reported that TLR-stimulated monocytes, which upregulate MICA, promoted IFN-γ release *via* interaction with NKG2D-expressing NK cells (59). In the presence of IL-12, NKG2DL-expressing monocytes were shown to not only stimulate IFN-γ release, but also to enhance antibody-dependent cellular cytotoxicity toward Ab-coated target cells (90). Results from the Davis lab indicated that NK cells and autologous human macrophages can engage two distinct types of interactions. On the one hand, low-dose LPS-stimulated macrophages can trigger NK cell proliferation, secretion of cytokines and increased killing of tumor targets *via* the 2B4/CD48 axis, while, on the other hand, macrophages activated with high doses of LPS expressed MICA and ULPBs, formed the so-called lytic synapse, and were lysed by NK cells *via* NKG2D (89). These data indicate that the activation status of macrophages can determine the outcome of their crosstalk with NK cells and that NKG2DL expression might be a signal for removal of activated macrophages to prevent exaggerated inflammation and tissue damage. In mice, activation of peritoneal macrophages with poly(I:C) induced Rae-1, H60, and MULT1 expression, along with IL-15, IL-18, and type I IFN production. These soluble factors could increase NKG2D expression on NK cells, leading to increased cytotoxicity in response to tumor cells expressing NKG2DLs. However, macrophages remained resistant to NK cell lysis due to the expression of Qa-1, a surface molecule engaging the inhibitory NK cell receptor NKG2A (91).

Besides a direct interaction that triggers immediate effector responses, such as cytokine production and cytotoxicity, myeloid cells expressing NKG2DLs can indirectly control the function of NKG2D expressed on effector lymphoid cells. It was shown that NKG2DL engagement can lead to NKG2D downregulation and that the constitutive presence of NKG2DLs can cause long-term desensitization of the NKG2D pathway (53, 92, 93). This phenomenon can be mediated by both membrane bound ligands and by ligands released in soluble form upon proteolytic shedding or *via* exosomes. While the effect of tumor-released soluble ligands/exosomes has been extensively studied, mainly as a mechanism of immune evasion, the contribution of myeloid cells to this phenomenon remains largely unrevealed. Only recently, a study showed that *in vivo* overexpression of Rae-1 on CD11c^high^ cells, comprising mainly DCs in mice, led to reduced NK cell-mediated cytotoxicity toward NKG2DL+ or MHC class I-deficient targets, compromising the ability of these animals to reject NKG2DL-expressing tumor cells, while the control of viral infection (MCMV) remained unaffected (94). Of note, continuous engagement of NKG2D was shown not only to affect NKG2D-dependent responses, but also to desensitize the signaling downstream of other activating receptors, such as NK1.1 and NKp46 (92, 95). Accordingly, Rae-1 expression on lymph node endothelial cells in steady state (53) or by myeloid cells in tumor-bearing animals (51) is responsible for NKG2D downregulaton and global desensitization of NK cells. Similarly, in cancer patients, the presence of MICA- and ULBP1-expressing myeloid cells in blood and tumor correlated with reduced NKG2D expression on NK cells (96). However, it will be important to further improve the mechanistic understanding of NKG2D downmodulation in disease conditions and to dissect whether NKG2D downregulation in patients truly results from the engagement with soluble and cell-expressing ligands or if other factors, such as TGF-β, might contribute in these settings.

In the past few years, ILCs, which mainly reside in tissues, have been identified as novel players in the regulation of tissue homeostasis, regeneration, and response to infection (80). Since helper ILC1 and ILC3 populations express NKG2D (6–8), investigating the importance of the NKG2D/NKG2DL axis in the putative crosstalk between ILCs and myeloid cells, particularly in tissues and/or pathologies where ILC populations play an important role, would be of high relevance.

## NKG2D Expression on T Cell Subsets: When and Where?

**Figure 2:**
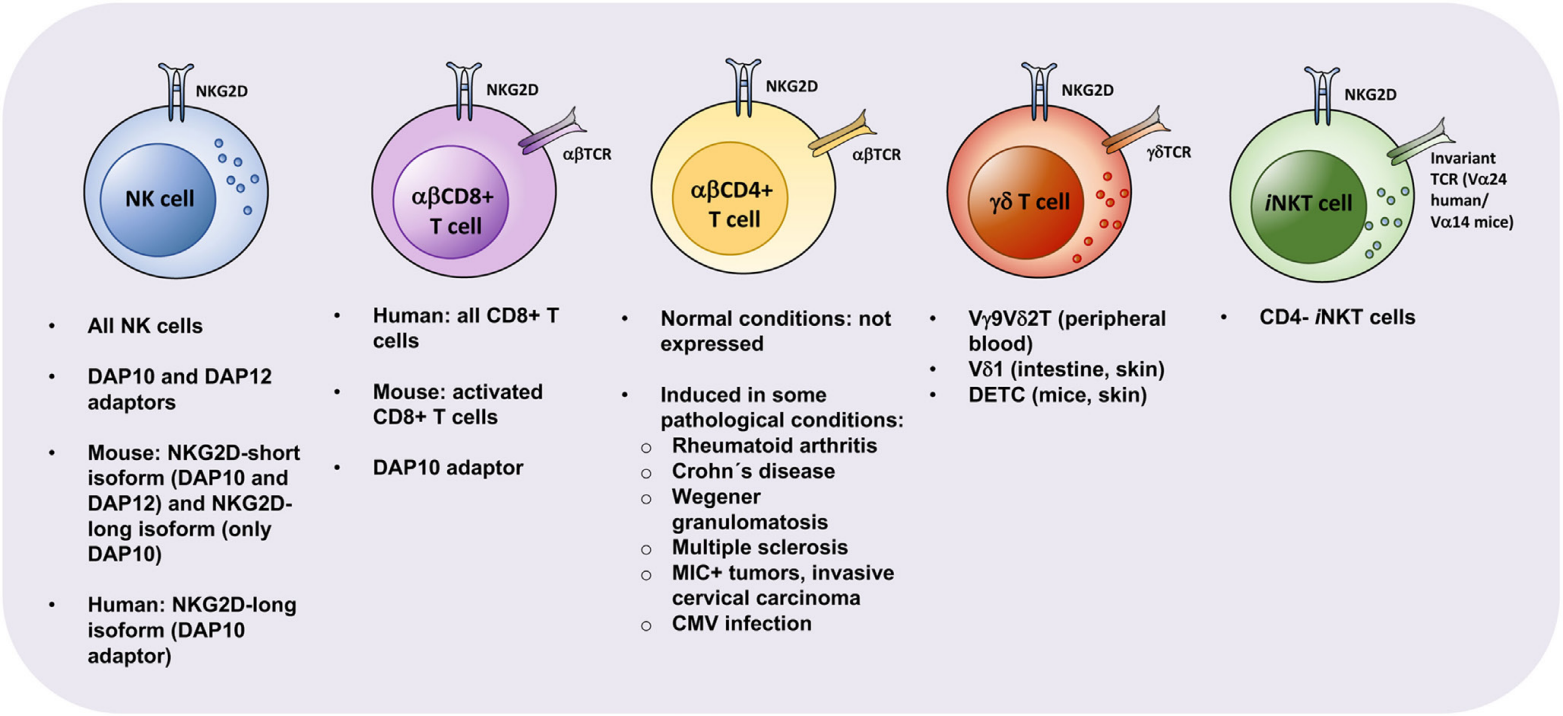
Properties of natural killer group 2, member D (NKG2D)-expressing lymphocytes. NKG2D can be expressed by natural killer (NK) cells, αβ CD8+ T cells, γδ T cells, invariant NKT (*i*NKT) cells, and αβ CD4+ T cells. However, its expression, alternative splicing, and coupling to adapter molecules vary between the indicated lymphoid subsets. Expression of NKG2D on certain lymphocytes correlates with activation (mouse αβ CD8+ T cells) or can be detected only in disease settings (αβ CD4+ T cells). While NK cells, *i*NKT cells and CD8+ T cells are circulatory and can be detected in peripheral blood, other lymphoid cells, such as helper innate lymphoid cells (data not shown) and certain γδ T cells, are tissue-resident and are involved in local tissue immune responses. Similarly, pathologic αβ CD4+ T cells are enriched in affected tissues in depicted autoimmune conditions. Abbreviations: DETCs, dendritic epidermal T cells; CMV, cytomegalovirus.

As referred above, NKG2D is a widely expressed receptor detectable on NK cells and several subsets of T cells, including CD8+ T cells, subsets of γδ T cells and NKT cells in steady state, and CD4+ T cells under certain pathological conditions. This transversal expression from innate to adaptive immune lymphocytes makes NKG2D a remarkable NK receptor. Thus, the focus on the dynamics of a NKG2D/NKG2DL axis should go beyond the NK cell-myeloid cell crosstalk. Below, we discuss the so far described NKG2D-expressing T cell subsets (see Figure 2).

**αβ CD8+ T Cells**. CD8+ T cells are the most representative T cell subset expressing NKG2D. While in mice expression of NKG2D is restricted to activated CD8+ T cells, in humans, all CD8+ T cells express NKG2D constitutively (97). In human, as with NK cells, CD8+ T cells do not express the short NKG2D isoform. In mice, although activated CD8+ T cells express both NKG2D isoforms, they usually lack expression of DAP12, contrarily to NK cells. Thus, for both species, NKG2D in CD8+ T cells seems to primarily signal *via* DAP10 (2, 3). It was reported that DAP12 could also be expressed by T cells and that, unlike NK cells, human activated CD8+ T cells required simultaneous signaling mediated by both DAP10 and DAP12 pathways (98). However, several studies support that DAP10 appears to be the most important adaptor for NKG2D signaling in CD8+ T cells (3, 22, 99).

In conventional CD8+ T cells, NKG2D has been shown to mainly serve as a costimulatory receptor for TCR-induced signaling (22, 69, 99–101). Although the costimulatory function of NKG2D was more evident for activated CD8+ T cells that lack CD28 (99, 100), it was also observed in naïve CD8+ T cells (101). In fact, the cytoplasmic domain of DAP10 comprises a signaling motif similar to CD28, which activates PI3K and leads to similar, though not identical effects on T cell costimulation (102, 103). Besides its role in decreasing the TCR threshold in CD8+ T cells, as a costimulatory receptor, several studies have shown that NKG2D can also work as an activating receptor *per se* on CD8+ T cells under certain conditions, namely upon prolonged exposure to IL-15 (104–106). In fact, IL-15 appears to be a key factor in arming the NKG2D-mediated cytolysis of effector CD8+ T cells. Meresse et al. showed that, in celiac disease, NKG2D expressed on intraepithelial intestinal lymphocytes could mediate direct cytolysis, putatively due to overexpression of IL-15 in this disease (104). Other studies demonstrated that prolonged exposure of human peripheral blood CD8+ T cells to IL-15 *in vitro* led to a functional NKG2D receptor *per se*, without the need of TCR coengagement for activation. IL-15, besides being involved in NKG2D and DAP10 induction and upregulation (104–108), was demonstrated to synergize with the NKG2D downstream signaling pathway through activation of PI3K, JNK, ERK, and cPLA2 (21, 104, 109, 110), thereby enabling NKG2D to mediate direct activation and cytolysis in a TCR-independent manner. Also, it was shown that NKG2D could enhance IL-15-mediated PI3K signaling in activated CD8+ T cells, promoting CD8+ T cell survival and memory formation (111), showing a bi-directional importance of the IL-15–NKG2D downstream signaling interaction. Moreover, NKG2D on CD8+ T cells was shown to rescue CD4-unhelped CD8+ T cell memory recall responses, but not effector responses, by repressing the transcription factor T-bet (112). Accordingly, besides activating CD8+ T cells and driving cytotoxicity, NKG2D has also been implied in the survival and memory formation of CD8+ T cells.

**αβ CD4+ T Cells**. Under physiological conditions, expression of NKG2D is not detectable on conventional αβCD4+ T cells. However, NKG2D-expressing CD4+ T cells have been described in human under certain pathological conditions. NKG2D+ CD4+ T cells were initially found on peripheral blood and synovial fluid from rheumatoid arthritis (RA) patients, putatively as result of increased TNF-α and IL-15 levels in this disease. Groh et al. showed that those cells promoted the cytotoxic damage against synoviocytes with anomalous expression of NKG2DLs (14). Later on, NKG2D+ CD4+ T cells have been associated with several other autoimmune diseases, such as Crohn’s disease (108, 113, 114), Wegener granulomatosis (115, 116), type 2 diabetes (117), multiple sclerosis (118), and systemic lupus erythematosus (SLE) (119, 120). Moreover, NKG2D+ CD4+ T cells accumulated in patients with MIC+ tumors (121) and cervical carcinoma (122–124). Also, NKG2D+ CD4+ T cells were identified in patients suffering from a human T cell lymphotropic virus type I-associated neurological disease (125) and linked with human cytomegalovirus infection (126).

Of note, NKG2D+ CD4+ T cells appear to have similar features to a previously described CD4+ CD28− T cell phenotype, which prevails in several pathologies, namely autoimmune disorders (127–132). In fact, NKG2D+ CD4+ T cells have been detected mainly within the CD4+ CD28− T cell population (14, 126, 133). Thus, previous studies describing the CD4+ CD28− T cell population should be revisited by addressing a putative role of NKG2D in disease progression and severity.

Similar to CD8+ T cells, NKG2D plays a major role as a costimulatory receptor, enhancing TCR-mediated responses in NKG2D+ CD4+ T cells. Several studies showed the involvement of this population in the pathology of autoimmune diseases contributing to disease progression or severity in an NKG2D-dependent manner. However, increased frequencies of NKG2D+ CD4+ T cells inversely correlated with disease activity in juvenile-onset SLE, suggesting that these T cells may also have regulatory effects (134). Moreover, a recent study showed that NKG2D+ CD4+ T cells were involved in Treg killing in an NKG2D–NKG2DL-dependent manner in SLE (120).

Besides the existence of mounting evidence about the association of NKG2D+ CD4+ T cells and pathological conditions, shown to correlate with an increase in NKG2DLs, the functional crosstalk between myeloid cells expressing NKG2DL and NKG2D+ CD4+ T cells remains mainly unaddressed.

**γδ T Cells**. In both humans and mice, γδ T cells comprise a small population of peripheral blood cells (around 2–5%), but are abundant in tissues, particularly in the intestine, reproductive tract, and skin (135–137). Vγ9Vδ2 T cells (also known as Vγ2Vδ2), are the most abundant population in human peripheral blood (50–95%) (138), while Vδ1 γδ T cells are mainly enriched in the intestine (together with Vδ3 γδ T cells) and in the skin. Both Vγ9Vδ2 and Vδ1 γδ T cell subsets are described to express NKG2D on their cell surface, associating with DAP10 for signal triggering. In human, the IL-17+ Vγ9Vδ2 T population was reported to lack the expression of NKG2D, although the majority of circulating Vγ9Vδ2 T cells expressed NKG2D receptor (139).

In 1998, Groh and colleagues described that MICA and MICB could be recognized by intestinal Vδ1 γδ T cells through their γδ TCR (140). Shortly after, the same group showed that the cell surface NKG2D expressed on intestinal Vδ1 γδ T cells recognized MICA, and that NKG2D–MICA engagement resulted in target cell recognition and killing, suggesting that NKG2D might function as a costimulatory receptor on γδ T cells (141). MICA was later confirmed to be directly recognized by Vδ1 T cells through their γδ TCR, although weakly compared to recognition by NKG2D (142). Several studies showed that Vδ1 γδ T cells can recognize NKG2DLs expressed on cancer cells, triggering their cytotoxicity against targets (143, 144).

Das and colleagues showed that infection with *M. tuberculosis* induced MICA on the surface of dendritic and epithelial cells, both *in vitro* and *in vivo* (145). Moreover, MICA engagement by NKG2D expressed on Vγ9Vδ2 T cells resulted in a considerable increase of the TCR-dependent Vγ9Vδ2 T cells response (145). In the absence of antigen, NKG2D+ Vγ9Vδ2 T cells did not lyse MICA+ targets, indicating that the NKG2D–MICA interaction is not sufficient to trigger Vγ9Vδ2 T cell-mediated lysis (145). By contrast, a subsequent study showed that NKG2D expressed on Vγ9Vδ2 T cell could induce by itself Vγ9Vδ2 T cell activation and NKG2D-dependent cytolysis of target cells (146). NKG2D has been shown to be involved in Vγ9Vδ2 T cell recognition of leukemia and lymphoma (147), as well as of solid tumors (148, 149). Similar to Vδ1 γδ T cell recognition of MICA, ULBP4 was described to bind to both NKG2D and γδ TCR of Vγ9Vδ2 T cells, mediating their activation and cytotoxicity (150). Thus, altogether those studies indicate that tumor-expressed NKG2DLs can be specifically recognized by both TCR and NKG2D expressed on human γδ T cells.

Dendritic epidermal T cells (DETCs) are epithelial γδ T cells that reside in murine skin. While it is described that only 25% of splenic γδ T cells and 5% of thymic γδ T cells express NKG2D, basically all DETCs are NKG2D+ in mice (69). So far, human counterparts of mouse skin γδ T cells with the same dendritic-like characteristics have not been identified. Girardi and colleagues have shown that DETCs can kill carcinoma cells in an NKG2D-dependent manner (151), providing the first evidence of NKG2D-mediated DETC activation. Afterward, it was found that DETCs displayed impaired wound healing properties upon NKG2DL blocking (65, 152) and, as well, that the NKG2D/NKG2DL interaction was involved in allergen-induced activation of DETCs in contact hypersensitivity (153).

As with the other γδ T cell subsets, whether NKG2D expressed on DETCs works as a costimulatory receptor or as an activation receptor by itself is still controversial (153–156). Ibusuki et al. showed that NKG2D engagement alone was sufficient to trigger degranulation, but not cytokine production, in DETCs, which was mainly mediated *via* the DAP10–PI3K-dependent signaling pathway (157). However, in this study, DETCs were expanded in culture with IL-2, which may account for the observed surpass of the need for TCR cotriggering. In fact, NKG2D could not trigger cytotoxicity in freshly *ex vivo* isolated DETCs (157). Of note, since it is known that cytokines, such as IL-15, can synergize with the NKG2D downstream signaling pathway, it is important to consider that the majority of protocols using γδ T cells are preceded by an expansion phase including IL-2 or IL-15 cytokines. This fact might account for controversial views of NKG2D as costimulatory or stimulatory receptor by itself in NKG2D-expressing populations.

***i*NKT Cells**. Invariant NKT cells represent a small population of blood cells (0.01–1% among peripheral blood T cells) in human that, however, can be found highly enriched in the liver, particularly in mice. Those cells express a semi-invariant TCR, characterized by Vα14-Jα18 in mice and Vα24-Jα18 in humans, which recognizes lipid-based antigens in the context of CD1d molecules. *i*NKT cells can be functionally distinguished by the expression of CD4 on the cell surface, both in human and mice. In general, CD4+ *i*NKT cells display a Th2-like profile, whereas CD4− *i*NKT are rather skewed toward Th1-like responses (158, 159). NKG2D surface expression is restricted mainly to the CD4 negative subset of *i*NKT cells (158, 160). Kuylenstierna and colleagues found that NKG2D stimulation in CD4− NKT cells could act as a costimulatory signal in response to suboptimal anti-CD3 triggering or CD1d-presented ligands. In the same study, NKG2D stimulation in CD4− NKT cells also mediated a direct NKG2D-dependent lysis of target cells, independent of invariant TCR engagement, thus demonstrating both a stimulatory and costimulatory role of NKG2D in those cells (160). Wang et al. showed that tumor-derived soluble MICs downregulated NKT cell NKG2D expression and consequently tumor cell killing *in vitro* (161), supporting an anti-tumor function of NKG2D+ NKT cells.

Besides playing a role in cancer, NKG2D+ NKT cells were shown to be enriched in certain pathologies, namely autoimmune diseases. Patients with type 2 diabetes showed increased NKG2D+ NKT cells in peripheral blood, when compared with healthy controls (162). Early onset SLE was associated with changes in the ratio of NKG2D/NKG2A expression in multiple cell types, including NKT cells (163). NKG2D+ NKT cells with a Th1-like profile were also increased in pre-eclampsia (164). Further studies focusing on the specific role of NKG2D expressed by *i*NKT cells, in both cancer and other pathologies, and on the potential crosstalk with myeloid cells *via* interaction with NKG2DLs, would be important.

Noteworthy, besides the existence of mounting evidence associating several subsets of NKG2D+ T cells and pathological conditions characterized by an increase in NKG2DLs, the functional crosstalk between myeloid cells expressing NKG2DL and NKG2D+ T cell subsets remains mainly unaddressed. In this regard, it would be important on the one hand, to determine the specific role of NKG2D in conditional knockout mice, where NKG2D could be selectively deleted on different cell populations; and on the other hand, to dissect in parallel the importance of the NKG2DL-expressing myeloid cells. Since myeloid cells are important APCs, the putative crosstalk between myeloid cells and T cells expressing NKG2D might be highly relevant. Particularly in pathological conditions, myeloid cells could not only trigger NKG2D expressed by T cells *via* NKG2DL but could also in parallel activate T cells *via* antigen presentation by MHC molecules, further enhancing or controlling specific T cell-mediated responses. As such, further studies focusing on the impact of NKG2DL expressed on myeloid cells in the crosstalk with T cells would be of major relevance.

Importantly, a connection between NKG2D, autoimmunity, and IL-15 is getting increasingly evident. First, several autoimmune diseases have been correlated with increased IL-15 cytokine levels (165, 166). Second, NKG2D induction, upregulation and its role in autoimmunity has been extensively demonstrated (167). Finally, a role of IL-15 in NKG2D upregulation and enhancement of downstream signaling has been shown. Interestingly, myeloid cells are widely regarded as main producers of IL-15, particularly under pathological conditions, trans-presenting IL-15 to responding cells. On the one hand, it is known that IL-15 is involved in NKG2DL upregulation (104, 110) and, on the other hand, it has been shown that this cytokine can upregulate or induce NKG2D expression (104–106, 110). In this regard, it is tempting to imagine a scenario where myeloid cells trans-present IL-15 to effector cells, leading to NKG2D induction and/or support of its signaling, while these cells at the same time trigger NKG2D *via* NKG2DL interaction.

## NKG2DLs and Myeloid Cells in Disease Settings

Multiple diseases are associated with NKG2DL upregulation, where its expression might be either protective or detrimental. Dysregulated receptor/ligand expression was reported in various autoimmune diseases, such as RA, colitis, celiac disease, multiple sclerosis, type 1 diabetes, or atherosclerosis, where their involvement was postulated to mainly promote inflammation (11–17, 104). By contrast, in conditions such as wound healing, although NKG2DLs were induced by initial tissue damage and cellular stress, NKG2D was shown to support tissue remodeling and regeneration (65). However, in many of the studied diseases and respective animal models, it remains unclear which cells express NKG2DLs in the affected tissues and what is the myeloid cell contribution to the overall ligand expression and NKG2D-driven responses.

In tumor-bearing mice, Rae-1 was detected on a subset of MDSCs and contributed to NK cell activation (67). In glioblastoma multiforme (GBM) patients, MICA and ULBP1 were detected on microglia, tumor-infiltrating myeloid cells, and circulating monocytes (96). In this study, the authors showed that lactate dehydrogenase isoform 5 (LDH5), secreted by tumor cells, was elevated in GBM patients’ sera and responsible for NKG2DL upregulation on healthy monocytes. *In vitro*, IL-2-activated NK cells degranulated, produced IFN-γ, and induced apoptosis of autologous NKG2DL-bearing monocytes. However, *in vivo*, LDH5-mediated induction of NKG2DLs might serve as a cancer immune evasion mechanism, as NK cells from GBM patients displayed reduced surface NKG2D expression and impaired function (96). Similarly, NKG2DL-expressing monocytes were detected in the blood of breast, prostate, and virus-induced liver cancer patients (96).

In *atherosclerotic plaques*, both endothelial cells and macrophages have been reported to express MICA/B (15). In line with that, exposure of monocyte-derived macrophages to acetylated low-density lipoproteins *in vitro* led to MICA/B induction (168). In a mouse model of atherosclerosis, Rae-1 expression was detected on macrophages, not only in plaques but also in the liver, which is affected by metabolic changes associated with disease (15). The liver is enriched in NKG2D-expressing ILCs and NKT cells, whose crosstalk with the myeloid compartment might play a significant role in atherosclerosis. Consistent with that hypothesis, it was shown that NKG2D-deficient animals displayed smaller plaques in aortas, reduced liver damage, and reduced levels of proinflammatory cytokines, cholesterol, and triglycerides in serum (15).

La Scaleia et al. reported increased NKG2DL expression in the colon mucosa of pediatric patients with active *inflammatory bowel disease* (IBD). Their results indicated that the NKG2DL+ cells displayed a macrophage-like morphological phenotype (17). In addition, in active ulcerative colitis, MICA and MICB expression was significantly upregulated in peripheral blood monocytes (17). MICA/B+ macrophages were also detected in the duodenal tissue of patients with celiac disease, where those ligands were distributed intracellularly in the form of cytoplasmic aggregates (169). These data suggest that the crosstalk of myeloid cells with NKG2D+ innate and adaptive lymphocytes might play a significant role in IBD-associated inflammation. Moreover, intracellular NKG2DLs might have a specific, so far unappreciated function, not only in myeloid cells, but also in enterocytes, where peri- and supra-nuclear NKG2DL aggregates were also detected (169).

In *experimental autoimmune encephalomyelitis*, a mouse model of multiple sclerosis, Rae-1δ and Rae-1γ were induced at mRNA and protein level in spinal cord early upon disease onset. Djelloul et al. demonstrated that myeloid cells, including macrophages and microglia, expressed both Rae-1 and MULT1 (170). Expression of these ligands correlated with myeloid cell recruitment to affected tissue and their proliferation. Furthermore, this study identified M-CSF as factor driving NKG2DL expression on microglia.

As in the diseases discussed above NKG2D-expressing lymphocytes play significant roles, displaying either protective or detrimental properties, and myeloid cells exert potent regulatory roles in their activation, it would be of great importance to determine the contribution of NKG2D to their interaction. Moreover, NKG2D seems to play a costimulatory role in lymphocyte activation, acting often in the context of the proinflammatory environment. As myeloid cells contribute to an inflammatory environment *via* both soluble factors and NKG2DLs, it is tempting to speculate that their therapeutic targeting in combination with existing treatments might help to reduce tissue damage, especially in context of autoimmune diseases.

## Conclusion and Future Perspectives

NKG2D is considered a major lymphocyte receptor detecting dysregulated cell homeostasis induced by infection or transformation. Distinct pathways activated by cellular stress can upregulate various NKG2DLs, thus conveying an alert-signal of potential cell dysfunction to the immune system. Importantly, NKG2DL upregulation is frequently accompanied by specific microenvironmental milieus that support NKG2D upregulation, NKG2D function, and NKG2DL induction. These milieus are characterized by the presence of proinflammatory cytokines, among which, IL-15 plays a central role. In tissues, tissue-resident and recruited NKG2D+ lymphocytes are crucial for detecting and eliminating infected and transformed cells. However, this function is greatly supported by the myeloid immune compartment. Similar mechanisms, leading to NKG2DL upregulation in infected and transformed cells, operate to induce their expression in myeloid cells as well. Thus, besides their classical functions, that include Ag presentation to T cells or cytokine-mediated activation of innate lymphocytes, myeloid cells use the NKG2D/NKG2DL axis to support a regulatory loop, leading to lymphocyte activation *via* NKG2D, which in turn can activate or eliminate myeloid cells. The NKG2D-mediated myeloid–lymphocyte interaction can have a dual effect on the lymphoid effector cell. On the one hand, it can lead to cell activation, which promotes cytotoxicity, survival, and/or cytokine production. On the other hand, it causes cell inactivation, as a consequence of ligand-induced receptor internalization and desensitization of activating pathways beyond NKG2D. Although some of the factors that regulate these processes are known, such as soluble ligands and chronic stimulation, it would be highly relevant to define molecular events that can shift NKG2D engagement toward activation, e.g., during anti-tumor responses, or inactivation in the case of autoimmunity.

In many experimental settings, especially in disease, the importance of the myeloid–lymphocyte activation/inactivation loop has not been fully addressed, namely due to the existence of a broad variety of NKG2DLs and due to their expression beyond the myeloid compartment, including stromal and parenchymal cells. Accordingly, it remains a major challenge to address the contribution of the myeloid compartment to NKG2D-dependent lymphocyte activation, especially in pathological settings. So far, conditional knockout animals lacking all NKG2DLs were not generated, but mice overexpressing Rae-1 ligands exist and their overexpression in specific cell types was obtained by the use of Cre-lox technology (94). However, the complexity of the NKG2DL system, concerning their differential expression, differential shedding susceptibility and the ability to induce NKG2D downregulation, might compromise the full understanding of complex pathologies. In addition, recent data indicating that the continuous engagement of the NKG2D receptor in steady state regulates its activity (53), suggest that the use of inducible systems of deletion or overexpression would be a better experimental choice.

Although the NKG2D-mediated crosstalk of myeloid cells and NK cells is relatively well understood, the outcome of the interaction of the different NKG2D-expressing T cell subsets with NKG2DL+ myeloid cells remain largely unknown. In many diseases, especially in autoimmune disorders, that are often associated with aberrant activation of myeloid cells, the presence of NKG2D-expressing T cells is well documented. It would be of great value to gain understanding of the relative contribution of NKG2D-expressing T cells in these settings and the involvement of NKG2D, especially for therapeutic targeting. For this purpose, mice carrying a conditional NKG2D deletion specifically in T cells would be valuable tools. In addition, novel populations of tissue-resident lymphocytes are emerging and their importance in regulating inflammation, tissue homeostasis, and regeneration is now eminent. Their topological position and fast responses to tissue damage, in cooperation with the myeloid compartment, along with the fact that NKG2DLs are the messengers of damage, impose them as attractive candidates that might utilize the NKG2D system to perform their functions. It is known that such scenarios operate in skin with tissue-resident γδ T cells being major players. Other barrier sites are awaiting further evaluation in the context of the NKG2DL/NKG2D crosstalk.

## Author Contributions

AS and MPC wrote the manuscript; MPC generated the figures; AC read the manuscript and provided critical input.

## Conflict of Interest Statement

AC is a member of the Scientific Advisory Board of Dragonfly Therapeutics.
